# Compact circularly polarized dual band antenna with modified patch for ISM and V2X communication systems

**DOI:** 10.1038/s41598-025-89937-7

**Published:** 2025-02-18

**Authors:** E. D. Kanmani Ruby, T. Sathiyapriya, P. Sundaravadivel, D. Rajesh Kumar, Om Prakash Kumar, Shweta Vincent, Pallavi R. Mane

**Affiliations:** 1 Department of Electronics and Communication Engineering, Vel Tech Rangarajan Dr. Sagunthala R & D Institute of Science and Technology, Chennai, India; 2 Department of Electronics and Communication Engineering, Dr Mahalingam College of Engineering and Technology, Pollachi, India; 3 Department of Artificial intelligence and machine learning, Saveetha Engineering College (Autonomous), Chennai, India; 4 Department of Electronics and Communication Engineering, Manipal Institute of Technology, Manipal Academy of Higher Education, Manipal, 576104 India; 5 Department of Mechatronics, Manipal Institute of Technology, Manipal Academy of Higher Education, Manipal, 576104 India

**Keywords:** Dual-band, Circular polarization, ISM, V2X, Compact, SDG 9, SDG 11, Engineering, Aerospace engineering, Electrical and electronic engineering

## Abstract

This paper introduces a novel design and development of a compact circularly polarized antenna operating at two distinct frequency bands, 2.5 GHz and 6 GHz. The innovation lies in using a modified circular patch and a uniquely designed feeding element, which optimize circular polarization performance while maintaining a low-profile form factor. Fabricated on an FR4 substrate with an overall thickness of 1.6 mm and dimensions of 62 mm × 62 mm × 1.6 mm, corresponding to approximately 0.5167λ × 0.5167λ × 0.0133λ at 2.5 GHz, the antenna achieves a compact configuration well-suited for space-constrained applications. Extensive simulations and measurements show close agreement, with axial ratios below 3 dB across both operational bands, ensuring effective circular polarization. The antenna achieves peak gains of 1.8 dBi at 2.5 GHz and 3.8 dBi at 6 GHz, highlighting its ability to combine compactness, dual-band functionality, and strong radiation characteristics. This proposed design provides a groundbreaking solution for Industrial, Scientific, and Medical (ISM) and Vehicle-to-Everything (V2X) communication systems, addressing the increasing demand for efficient, dual-band, and space-saving wireless communication solutions.

## Introduction

Recent advancements in dual-band circularly polarized (CP) antennas have significantly addressed the increasing demand for compact, efficient, and versatile designs. In 2023 and 2024, researchers proposed a variety of innovative solutions for applications such as satellite communications, wearable devices, IoT, RFID, and medical technologies. These developments prioritize improved performance metrics like bandwidth, gain, and axial ratio, while ensuring compact size and reliable operation.Key contributions include a dual-band dual-CP transmit-array antenna tailored for satellite systems. This design employs a novel four-color scheme for multibeam generation and delivers excellent gain and axial ratio across two bands, highlighting its suitability for high-performance satellite communications^1^. Another notable innovation is an ultrathin CP antenna for mobile devices, achieving dual-band operation between 6.38 and 8.21 GHz through parasitic patch integration and via fences, offering a compact and efficient solution with stable CP characteristics^2^. For wearable technology, a flexible dual-band CP antenna has been developed with a low-profile design, providing effective operation across two frequency bands for body-worn applications^3^. In RFID systems, a dual-sense CP antenna featuring crossed dipoles operates at 915 MHz and 2.45 GHz, achieving excellent isolation and high radiation efficiency, making it ideal for reader applications^4^. In satellite communications, a stacked patch antenna supports dual-band CP operation in K- and Ka-bands with both left- and right-hand polarization, meeting stringent modern system requirements^5^. In the medical field, an implantable dual-band CP antenna optimized for wireless power transfer and data telemetry operates at 915 MHz and 2.45 GHz, using modified square patches to ensure compactness and biocompatibility^6^. Additionally, a slot antenna for sub-6 GHz applications integrates advanced feed structures to enhance gain and CP stability, supporting IoT and Wi-Fi devices^7^. Other breakthroughs include a frequency-ratio tunable CP antenna utilizing parasitic elements for versatile IoT applications^8^, a dual-mode CP antenna enabling independent polarization control across two bands for adaptable communication protocols, and a metasurface-based CP antenna that delivers dual-band operation with enhanced bandwidth and efficiency, making it ideal for next-generation satellite systems^9^.The works^10–12^ focus on advanced wearable and textile antennas with dual-band and circular polarization features. Oshi et al. designed a dual-band, dual-sense textile antenna with AMC backing for GPS and WBAN/WLAN applications. Yang et al. developed a dual-band textile antenna with dual circular polarizations using polarization rotation AMC for off-body communications. Dong et al. introduced a novel dual-band circularly polarized wearable antenna. Together, these studies emphasize compact, flexible, and multifunctional antenna designs for wearable technologies. These recent advancements underscore a strong emphasis on compact, high-performance designs tailored to the demands of modern wireless communication systems. They highlight the potential of dual-band circularly polarized (CP) antennas to meet diverse application needs while ensuring efficient and reliable operation. CP antennas are particularly valued for their versatility across a wide range of applications, including radio-frequency identification (RFID), radar systems, and spacecraft, especially in global navigation satellite systems (GNSS). Their key advantages include resistance to orientation constraints^13^ and the ability to reject multipath signals, enhancing polarization efficiency^14^. Various CP antenna designs have been developed to achieve a wide axial ratio bandwidth (ARBW), crucial for high channel capacity in modern wireless systems. Examples include slot antennas with L-shaped feedlines^15,16^, vertical L-shaped ground planes^17,18^, cross dipoles with phase delay lines^19^, and asymmetric T-strips fed by coplanar waveguides^20^. Monopole antennas^21–23^ are also widely used for wideband CP applications. However, approaches like dual-feed systems or multiple substrates, though effective in increasing bandwidth, often introduce greater complexity and higher costs^24–26^. Simpler, low-profile single-feed slot antennas are easier to manufacture but are typically limited in bandwidth and gain due to their bidirectional radiation pattern. To improve directivity, metal reflectors are often placed a quarter wavelength from the antenna, but this can reduce bandwidth^27^. Addressing these limitations, the concept of integrated filter antennas has gained traction, employing electromagnetic (EM) coupling to expand the bandwidth of single-feed CP antennas. Recent research has renewed interest in this approach, particularly in designs incorporating linearly polarized components^28,29^. Additionally, CP antennas with built-in filters are becoming more prevalent. In these designs, the feedline may integrate the filter^30,31^, or the resonant radiating element may serve as the final resonator^32–34^. These innovations represent significant progress in the quest for efficient, cost-effective CP antenna solutions.

The works of Hussain et al.^36–38^. present significant contributions to advanced antenna and electromagnetic wave technologies. In^36^, they introduced a miniature broadband electromagnetic wave absorber optimized for X-band signals, showcasing effective absorption suitable for applications like stealth and radar systems. In^37,38^, they proposed a miniaturized arrow-shaped flexible filter-embedded antenna designed for industrial and medical applications, achieving compactness and high performance. Additionally, they developed a compact PDMS-based ultra-wideband antenna for wireless body area networks, emphasizing flexibility, biocompatibility, and wide operational bandwidth, making it ideal for wearable and healthcare applications. These studies collectively highlight innovative approaches to miniaturization, flexibility, and application-specific designs, advancing the field of modern antenna systems.

The studies by Wang et al.^39^ and Abbas et al.^40^ contribute significantly to advancements in antenna design and reconfigurable technologies. Wang et al. proposed a novel method for hybridizing folded transmit arrays with Fabry-Pérot cavities, resulting in a low-profile antenna capable of flexible frequency design, making it suitable for compact and efficient applications. Abbas et al. introduced an electronically reconfigurable ultra-wideband antenna with a highly selective stop-band. Utilizing electromagnetic bandgaps and PIN diodes, their design enables precise frequency control and adaptability to dynamic spectral requirements. Together, these works highlight innovative approaches to improving antenna performance, compactness, and reconfigurability for diverse use cases.

An antenna design^41^ that incorporates a surface-mounted artificial magnetic conductor (AMC) to enable gain reconfiguration. This adaptive feature allows the antenna to effectively mitigate high-intensity radiated field (HIRF) interference, making it ideal for use in environments with sensitive electronics.

Filters and reflective intelligent surfaces (RIS) significantly enhance V2X communication systems by addressing critical challenges such as interference, adaptability, and signal quality. Filters, as highlighted by Wen et al.^46^, provide wideband coverage and interference management through tunable bandpass designs with multiple transmission zeros, ensuring reliable operation across diverse V2X frequency ranges. Meanwhile, RIS technologies, such as those explored by Wu and Ismail^47^ and Hongyun et al.^48^, enable dynamic control of signal propagation by leveraging joint active and passive beamforming. This capability improves channel quality and mitigates environmental challenges, such as obstructions in urban settings. Together, these advancements optimize spectral efficiency and communication reliability, addressing the stringent requirements of V2X applications.

From the literature it is noticed that circularly polarized (CP) antennas are widely used in various applications due to their ability to reject multipath signals and maintain polarization efficiency under different orientations. However, many CP antenna designs face limitations, particularly in achieving wide axial ratio bandwidth (ARBW) while maintaining low fabrication complexity and cost. Techniques such as dual-feed systems and multiple substrates improve bandwidth, but they increase design complexity. Similarly, single-feed slot antennas, despite being simpler and lower-profile, have limited bandwidth and gain. Existing solutions involving integrated filter antennas to enhance bandwidth have gained attention, but they often focus on antennas using linearly polarized components, and incorporating filters into the CP antenna designs remains challenging. Thus, there is a need for compact, dual-band CP antennas that provide wide ARBW, strong gain, and simplified fabrication processes for modern wireless communication systems.

This paper addresses the identified research gap by presenting a compact circularly polarized antenna that operates at two distinct frequency bands, 2.5 GHz and 6 GHz. The antenna utilizes a modified circular patch and a novel feeding element, simplifying the design and reducing complexity. With an overall dimension of 62 mm × 62 mm × 1.6 mm, it maintains a low profile, suitable for space-constrained applications. The proposed antenna achieves axial ratios below 3 dB across both frequency bands, indicating efficient circular polarization. Furthermore, it has sufficient gain (1.8 dBi at 2.5 GHz and 3.8 dBi at 6 GHz), is still small, and can work with both bands, making it a reliable choice for modern wireless systems that require simple, dual-band CP antennas.

A few major contributions of the work are summarized as follows.

1. Dual-Band Capability: Works at two frequencies—2.5 GHz and 6 GHz.
2. Simple, Compact Design: Uses a modified circular patch and a new feeding element, making it easy to build with reduced complexity.
3. Space-Efficient: Small size (62 mm × 62 mm × 1.6 mm), suitable for space-limited applications.
4. Efficient Polarization: Achieves circular polarization with axial ratios below 3 dB at both frequency bands.
5. Good Performance: Delivers sufficient gain (1.8 dBi at 2.5 GHz and 3.8 dBi at 6 GHz).
6. Versatile Application: Fits well for modern wireless systems needing dual-band, circularly polarized antennas.

The entire article is subdivided into six sections. Section I introduces the Antenna Geometry and Analysis, describing the modified circular patch design and innovative feeding element that enables dual-band operation. Section II presents a Parametric Study exploring how variations in design parameters impact performance. Section III analyzes the Surface Current Distribution at 2.5 GHz and 6 GHz to explain the antenna’s circular polarization behavior. Section IV discusses Measured Results, comparing key performance metrics like reflection coefficient, axial ratio, and gain against simulated results. Section V provides a Performance Comparison, showcasing the proposed antenna’s advantages over existing designs in size and efficiency. Finally, Section VI covers the potential application of antenna and investigates how different installation environments affect the antenna’s reliability in real-world scenarios.

## Antenna geometry and analysis

**Fig. 1:**
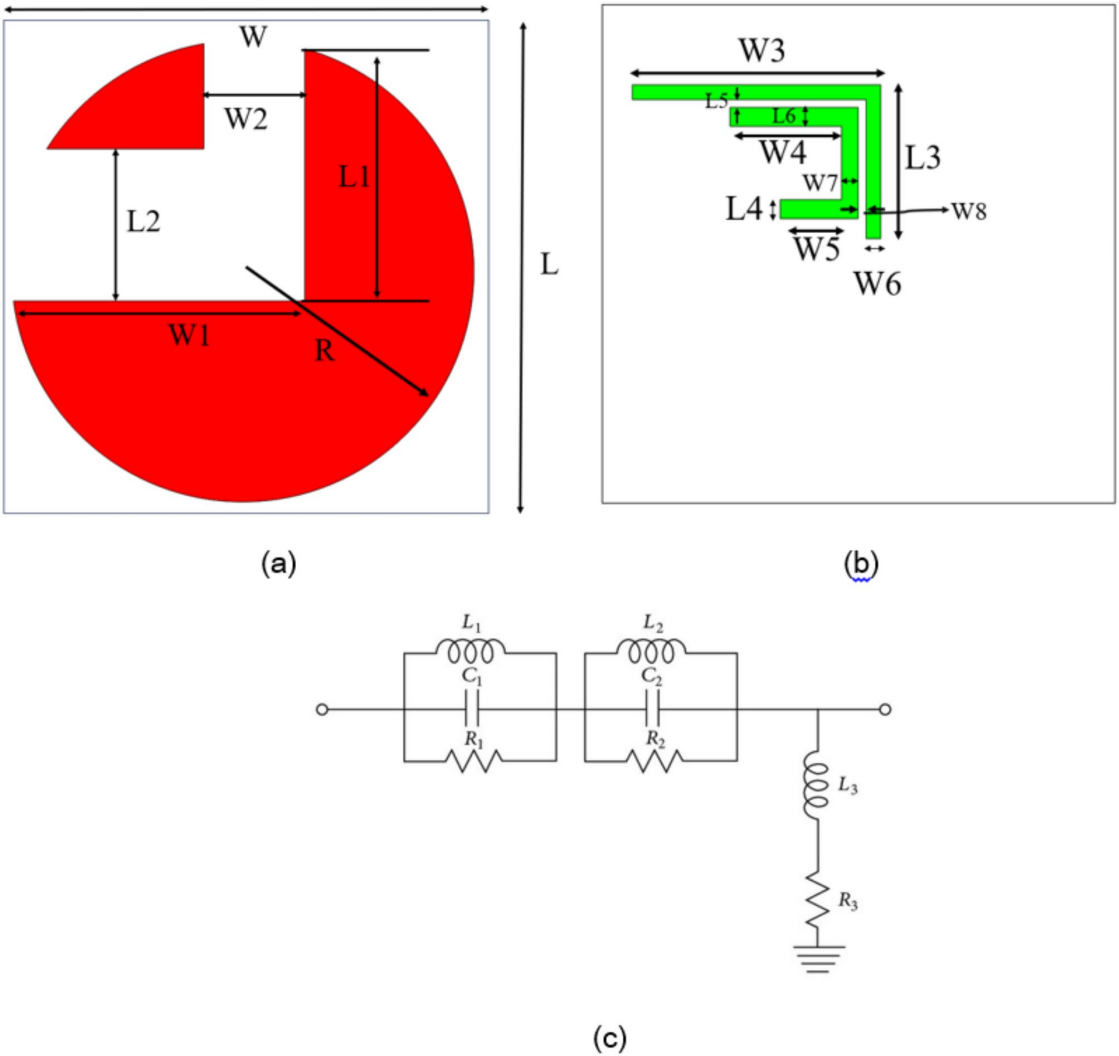
(**a**) Front view, (**b**) Back view of the proposed antenna, (**c**) equivalent circuit of the proposed antenna.

Figure 1a, b illustrate the design of a circularly polarized antenna, providing both front and back views with detailed dimensions. The front view consists of a semicircular patch antenna enclosed within a square substrate with a side length of L. The patch has a radius R and is critical to achieving circular polarization. The overall width of the substrate is denoted as W. A rectangular slot is etched out from the semicircular patch, with its width and length specified as W2 and L2, respectively. This slot is designed to perturb the current distribution, which is essential for generating circular polarization. Additionally, W1 and L1 are the dimensions that further define the geometry of the patch, influencing the antenna’s resonant frequency and axial ratio.

The back view presents a microstrip feed network for feeding the antenna with the correct phase and amplitude distribution to achieve circular polarization. The feeding element comprises several segments, each with specific dimensions: W3, W4, W5, W6, and W7 represent the widths of the microstrip lines, while L3, L4, L5, and L6 correspond to their respective lengths. These parameters are critical in determining the impedance matching, phase shift, and overall performance of the antenna in generating circularly polarized radiation. The antenna’s circular polarization is achieved through careful patch and feed network design, ensuring that the orthogonal modes are excited with a 90-degree phase difference. Table 1 presents the various design parameters of the proposed antenna. The theoretical calculations of the proposed antenna are characterized from^1–4^. For a circular patch antenna, the resonant frequency $\:{f}_{r}$ is given by^43^,

1 $$\:{f}_{r}=\frac{1.8412\:c}{2\pi\:a\sqrt{{\xi\:}_{r}}}$$

Where:$\:{f}_{r}$ = resonant frequency

$\:c$ = speed of light in vacuum (3 × 10^8m/sec)

$\:a$ = effective radius of the patch (mm)

$\:{\xi\:}_{r}$ = relative permittivity (dielectric constant) of the substrate

Due to fringing fields, the effective radius $\:{a}_{eff}$ is slightly larger than the physical radius aaa $\:a$. The effective radius is calculated as:

2 $$a_{{eff}} = a\left[ {1 + \frac{{2h}}{{\pi a\xi _{r} }}\left( {\ln (\frac{{\pi a}}{{2h}}} \right) + 1.7726} \right]^{2}$$

Where:

$\:a$ = physical radius of the patch (mm)

$\:h$ = height (thickness) of the substrate (m)

he physical radius aaa of the circular patch can be determined approximately using the following equation for a given resonant frequency:

3 $$\:a=\frac{F}{2\pi\:f\sqrt{{\xi\:}_{r}}}$$

Where $\:F$ is a constant related to the resonant mode, typically given by:

4 $$\:F=8.719\:{\rm\:X}\:{10}^{9}$$

**Table 1:** Various parameters of proposed antenna.

Parameters	L	L1	L2	L3	L4	L5	L6	*R*
Values (mm)	32	32.871	19.8141	18.3234	2.2536	0.9349	1.7563	31
Parameters	W	W1	W2	W3	W4	W5	W6	W7
Values (mm)	32	37.8516	13.1211	29.5526	13.2537	7.2895	1.7564	1.9883

The resonant theory is essential for understanding the operation of antennas at specific frequencies. It explains how an antenna achieves resonance by matching its physical dimensions and electrical properties to the wavelength of the operating frequency, minimizing energy loss and maximizing efficiency. The equivalent circuit model^42^ is a valuable tool for providing deeper insight. In this model, the antenna is represented as a combination of reactive components—inductors (L), capacitors (C), and resistors (R)—that mimic its behaviour. For an antenna operating at 2.5 GHz and 6 GHz, the equivalent circuit includes inductance for magnetic field effects, capacitance for the electric field, and resistance to represent radiation and loss. At resonance, the reactances of the inductor and capacitor cancel each other out, leaving only the resistive component, which corresponds to the radiated power. The model helps as in Fig. 1(c) visualize how the antenna impedance matches the source or load at these frequencies, enabling optimal performance and efficient power transfer. Table 2 listed various passive components of the antenna’s equivalent circuit.

**Table 2:** Various parameters of proposed antenna.

Components	L1	C1	L2	C2	R1 and R2	L3	R3
Values	4.05 nH	1pF	0.7 nH	1pF	50Ω	0.2 nH	1KΩ

## Parametric study and design evolution

**Fig. 2:**
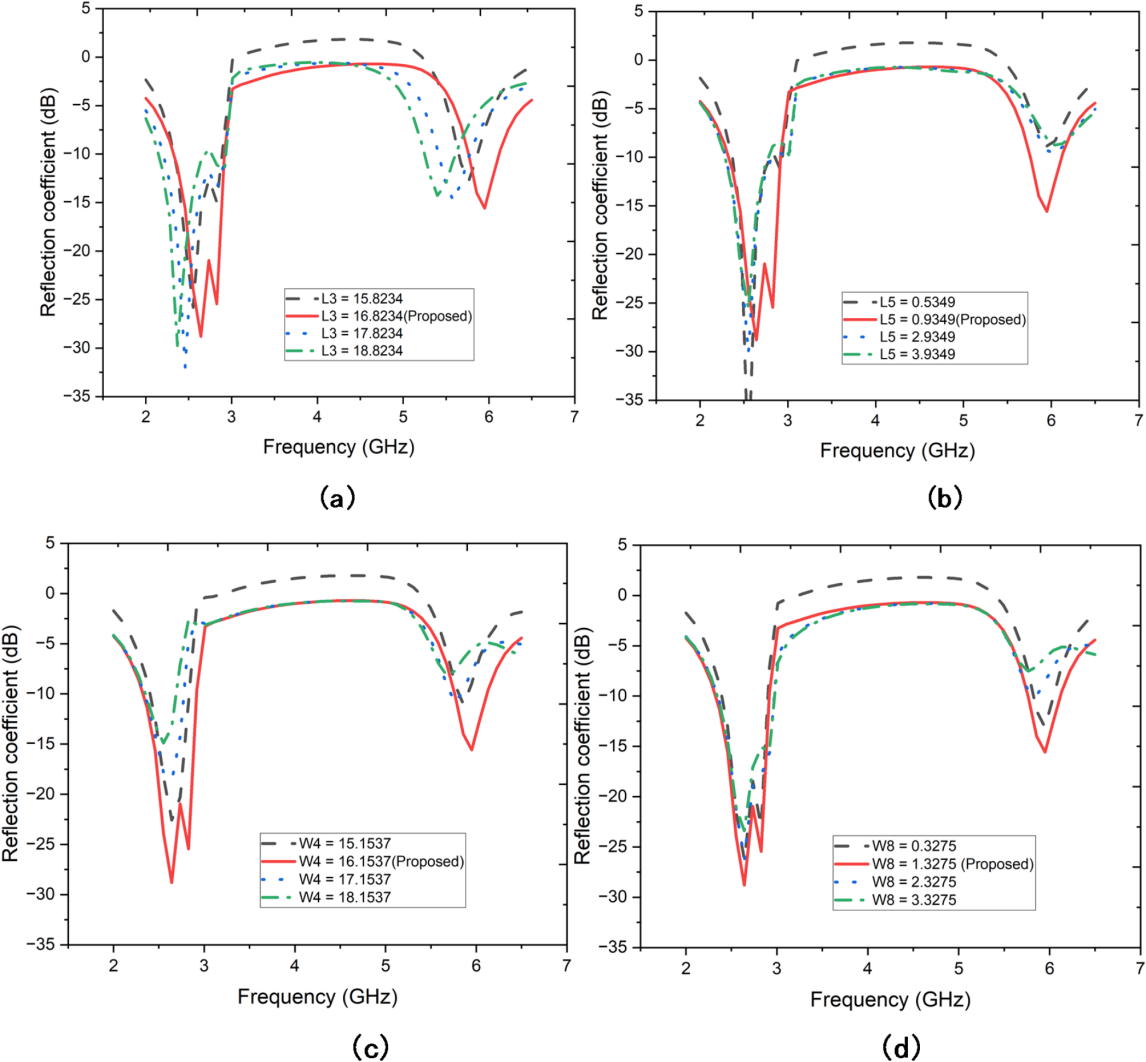
Parametric analysis when altering (**a**) L-shaped feed length (L3), (**b**) distance between L and C-shaped feeding structure (L5), (**c**) Length of the C-shaped feeder (W4), (**d**) distance between L and C -shaped feeding structure (W8).

This parametric study provides in-depth insights into the sensitivity of the proposed antenna design to variations in key geometrical parameters. Figure 2(a), investigates how varying the length of the L-shaped feed (L3) influences the antenna’s reflection coefficient. Four different lengths are examined: 16.8234 mm (proposed), 17.8234 mm, 18.8234 mm, and 19.8234 mm. The results show that altering L3 has a noticeable effect on both the resonant frequency and the bandwidth of the antenna. As L3 increases, there is a noticeable shift in the resonant frequency towards lower frequencies, accompanied by a slight degradation in the reflection coefficient. The proposed length of 16.8234 mm achieves the deepest nulls in S11, particularly around 2.5 GHz and 6 GHz, which are the primary target bands. This suggests that L3 plays a critical role in tuning the antenna’s resonance and ensuring adequate bandwidth for the intended applications.

Figure 2(b) explores the effect of varying the distance (L5) between the L-shaped feed and the T-shaped feeding structure. The distances considered are 0.9349 mm (proposed), 1.9349 mm, 2.9349 mm, and 3.9349 mm. The analysis reveals that L5 has a significant influence on the antenna’s impedance matching and resonant frequency. Specifically, increasing L5 causes the resonant frequencies to shift lower and broadens the bandwidth slightly. However, this comes at the cost of higher S11 values, indicating less efficient power transfer. The proposed distance of 0.9349 mm offers the most optimal impedance match, with deep S11 nulls at the desired frequencies, confirming that this parameter is crucial for maintaining effective coupling between the feeding structures. Figure 2(c) examines the effect of varying the length of the C-shaped feeder (W4) on the antenna’s performance. The lengths analyzed are 16.1537 mm (proposed), 17.1537 mm, 18.1537 mm, and 19.1537 mm. The results demonstrate that changes in W4 primarily affect the resonant frequencies and the sharpness of the S11 nulls. As W4 increases, there is a downward shift in the resonant frequency, with a corresponding reduction in the sharpness of the reflection coefficient nulls. The proposed length of 16.1537 mm provides the best trade-off between bandwidth and resonance, with deep nulls centered around the target frequencies, ensuring efficient radiation and minimal reflection. Figure 2(d) focuses on the distance (W8) between the L-shaped feed and the C-shaped feeding structure. The distances considered are 1.3275 mm (proposed), 2.3275 mm, 3.3275 mm, and 4.3275 mm. The analysis shows that W8 has an obvious impact on both the resonant frequencies and the overall impedance matching of the antenna. As W8 increases, the resonant frequency shifts downward, and the bandwidth slightly increases. However, the S11 values rise, particularly in the higher frequency bands, indicating a reduction in the antenna’s efficiency. The proposed distance of 1.3275 mm achieves the lowest S11 values across the target bands, with deep nulls that ensure efficient power transfer and minimal reflection.

**Fig. 3:**
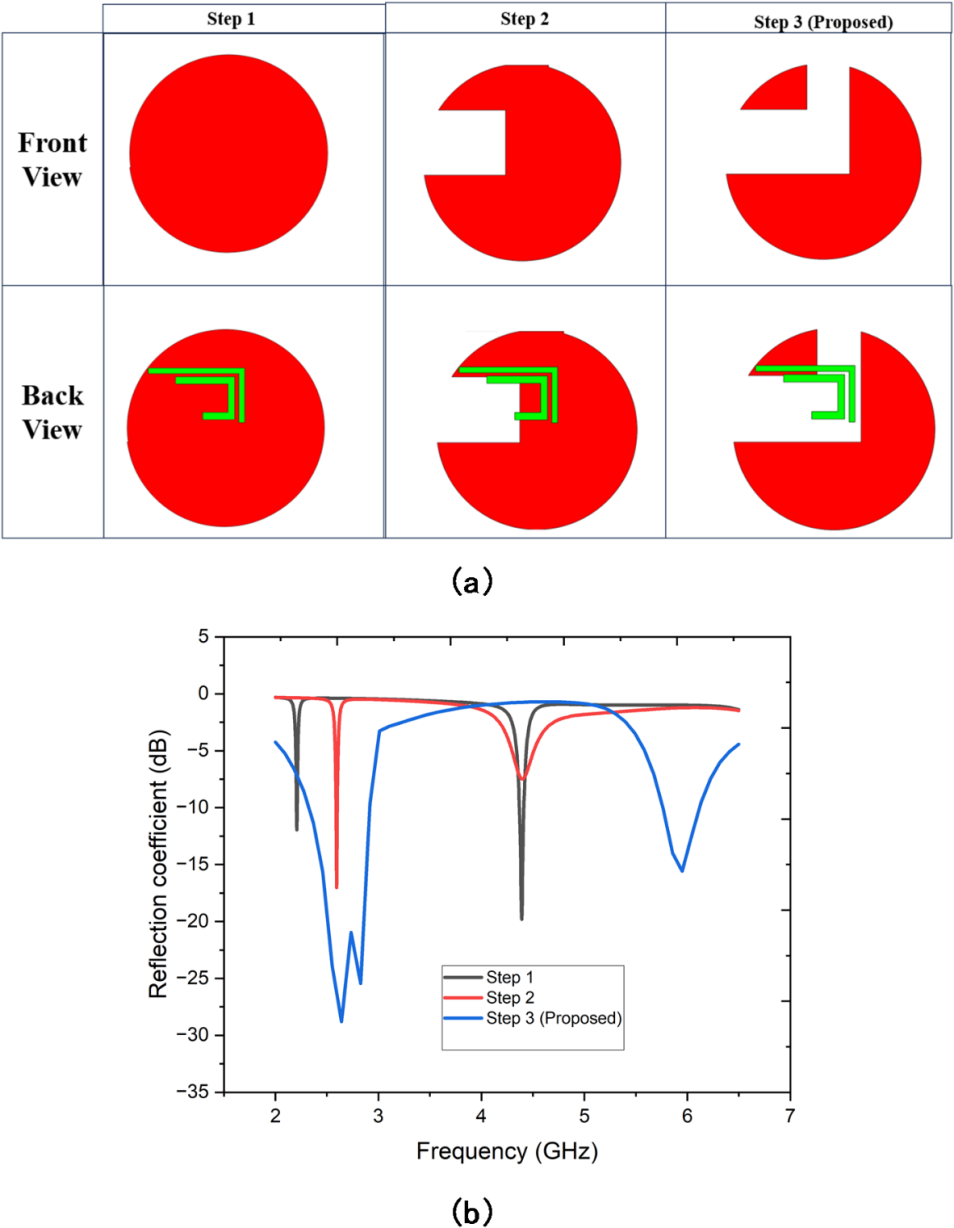
(**a**) Design evolution of the proposed antenna and their (**b**) reflection coefficient.

Figure 3 illustrates the design evolution of a proposed antenna and its corresponding reflection coefficient performance. In Fig. 3(a), the antenna design is depicted across three stages (Step 1, Step 2, and Step 3) from both the front and back views. Step 1 starts with a simple circular structure, while subsequent steps incorporate modifications, such as cutouts and the addition of a green conductive trace (feeding strip), to improve performance. These iterative design adjustments culminate in the proposed Step 3, which likely optimizes the antenna’s geometry for specific performance goals. In Fig. 3(b), the reflection coefficient (S11) is plotted against frequency for each design step. The curves demonstrate a clear progression in performance, where Step 3 exhibits deeper and broader reflection coefficient minima compared to the earlier steps, indicating improved impedance matching and enhanced bandwidth. The optimized Step 3 design appears to target multiple frequency bands with a reflection coefficient below − 10 dB, which is indicative of efficient signal radiation and reception across these frequencies. This evolution highlights the effectiveness of the design modifications in achieving the desired operational characteristics.

## Surface current distribution

**Fig. 4:**
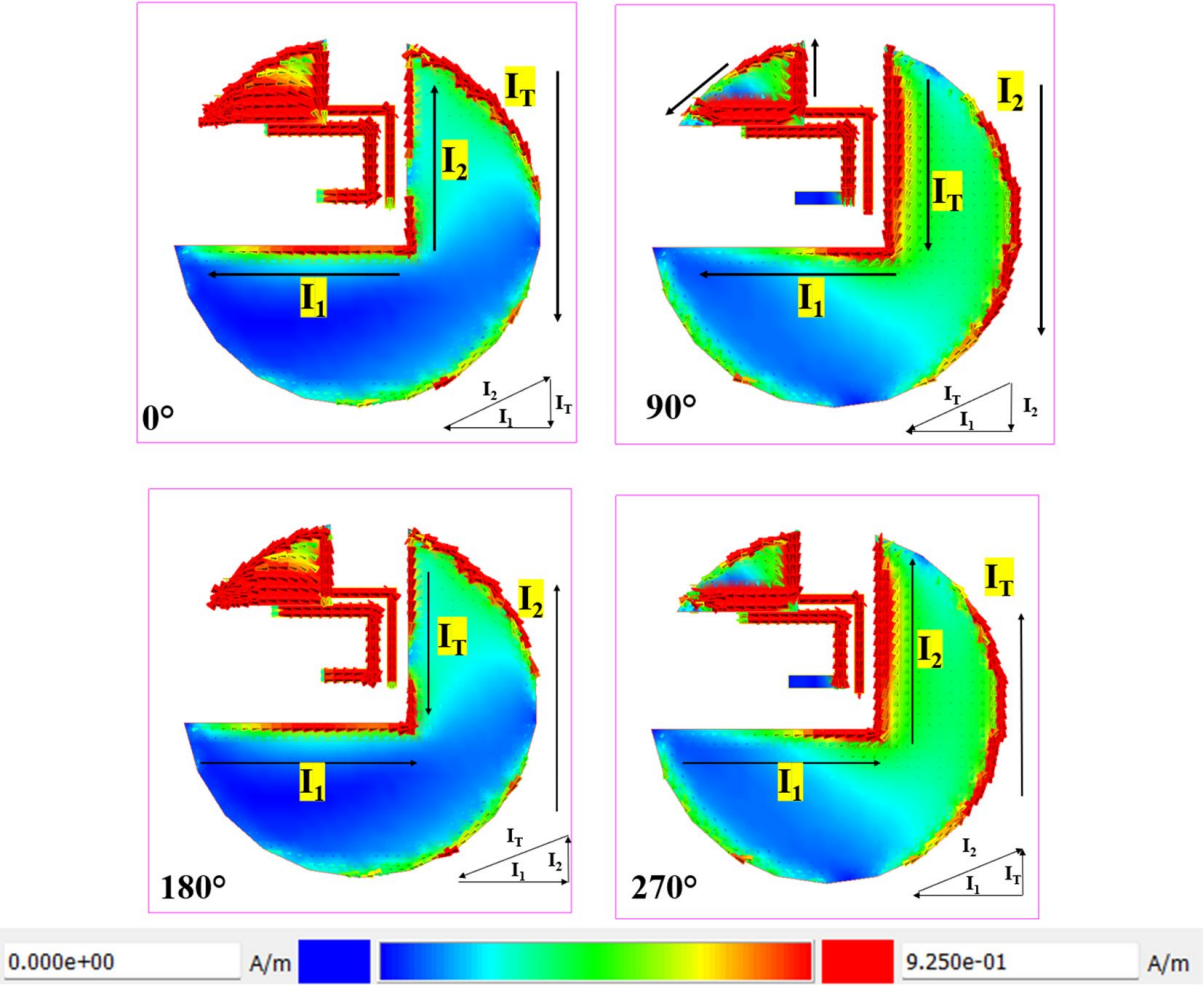
Surface current distribution at 2.5 GHz.

The Fig. 4 shows the surface current distribution of a circularly polarized (CP) antenna at 2.5 GHz for four phase angles: 0°, 90°, 180°, and 270°. At 0°, the current (`I₁`) is concentrated along the lower left, with the current vectors showing strong intensity near the feed point. As the phase progresses to 90°, the current rotates 90° clockwise, with high intensity shifting to the lower right quadrant, confirming the rotational nature essential for circular polarization. At 180°, the current distribution mirrors the 0° phase but in the opposite direction, with `I₁` still dominant. By 270°, the current rotates another 90°, aligning with the 90° phase but in reverse. This smooth rotation of the surface current across the antenna structure confirms right-hand circular polarization (RHCP), making the antenna effective for robust signal reception in wireless communication systems.

**Fig. 5:**
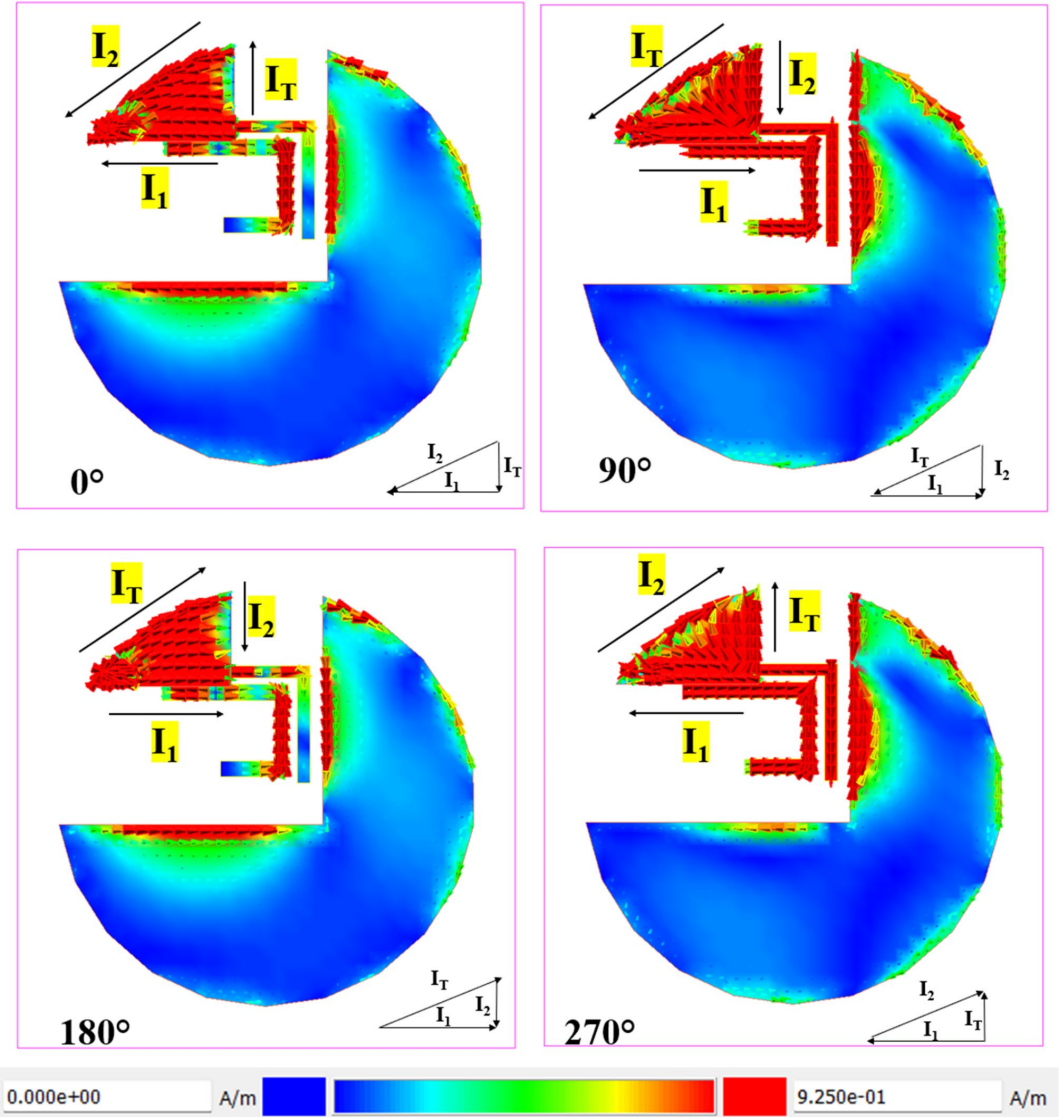
Surface current distribution at 6 GHz.

Figure 5 shows the surface current distribution of a circularly polarized (CP) antenna at 5.5 GHz across four phase angles: 0°, 90°, 180°, and 270°, which demonstrates the antenna’s capability to achieve right-hand circular polarization (RHCP). At 0°, the current (`I₁`) is highly concentrated along the lower left, with significant intensity near the feed line. At the same time, the upper arm (`I₂`) shows moderate current, indicating an asymmetric current distribution critical for initiating circular polarization. As the phase advances to 90°, the current vectors rotate 90° clockwise, shifting the high-intensity region to the lower right quadrant, illustrating the rotational behaviour essential for CP operation. At 180°, the current distribution mirrors the 0° phase but with reversed flow directions, maintaining the rotational symmetry necessary for RHCP. By 270°, the current completes another 90° rotation, with the positions of `I₁` and `I₂` swapped, confirming a consistent phase progression.

## Measured results and discussions

**Fig. 6:**
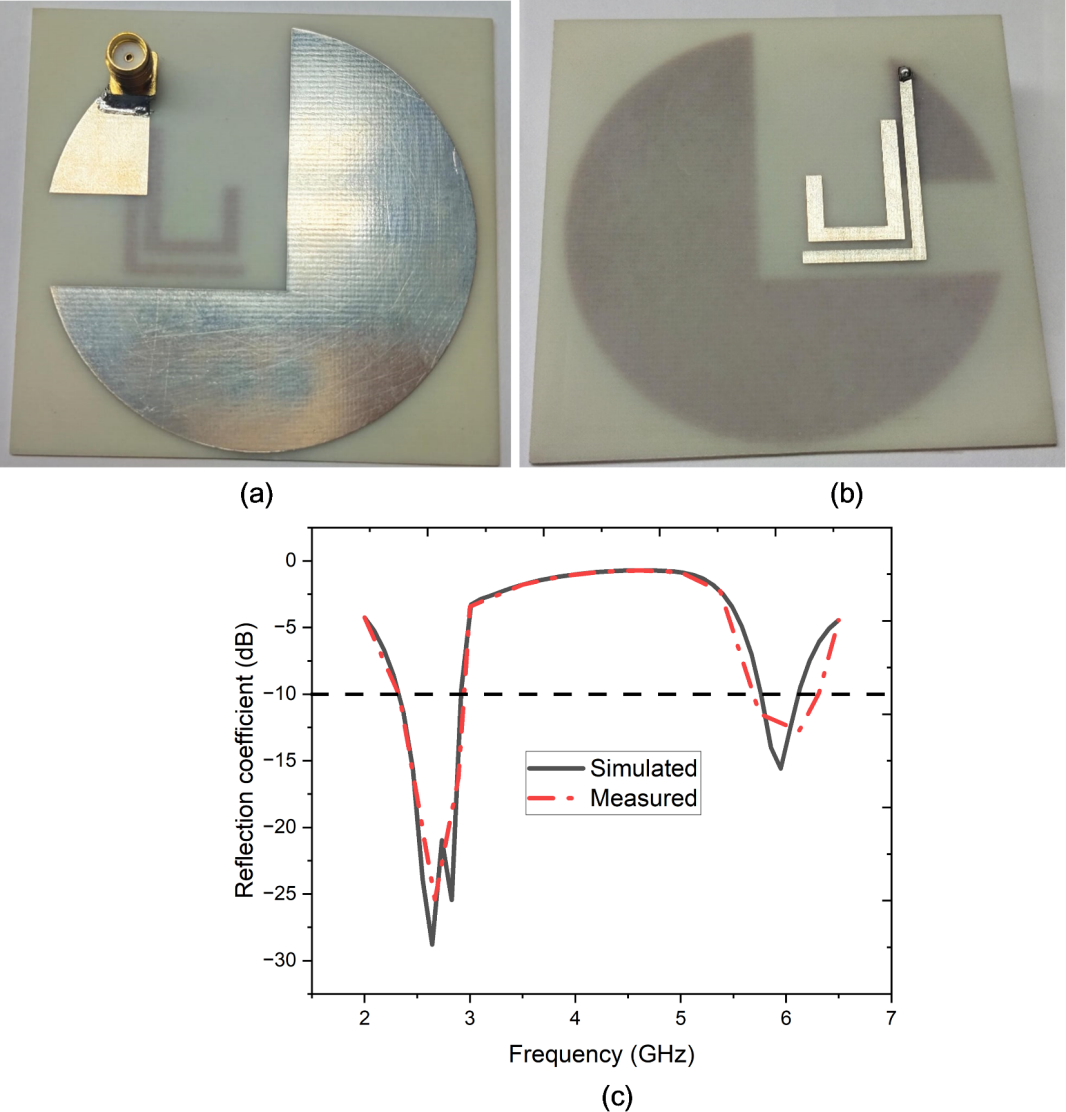
Fabricated prototype of the proposed antenna (**a**) front view, (**b**) view and (**c**) simulated and measured reflection coefficient.

The fabricated prototype of the proposed antenna is depicted in Fig. 6(a) and (b). Figure 6(c) reveals two distinct frequency bands around 2.5 GHz and 6 GHz where the reflection coefficient dips significantly below − 10 dB, indicating strong impedance matching and efficient radiation at these frequencies. The simulated and measured curves closely align, showing deep nulls at approximately 2.5 GHz and 6 GHz, with reflection coefficients reaching as low as -30 dB and − 16 dB, respectively. This close agreement between simulation and measurement across the operational bands confirms the antenna’s effectiveness, particularly in minimizing reflection and maximizing power transfer at these critical frequencies. The slight variations between simulated and measured results may be due to practical measurement conditions or slight differences in the physical realization of the antenna, but overall, the antenna performs well within the desired frequency ranges, ensuring efficient operation in those bands.

**Fig. 7:**
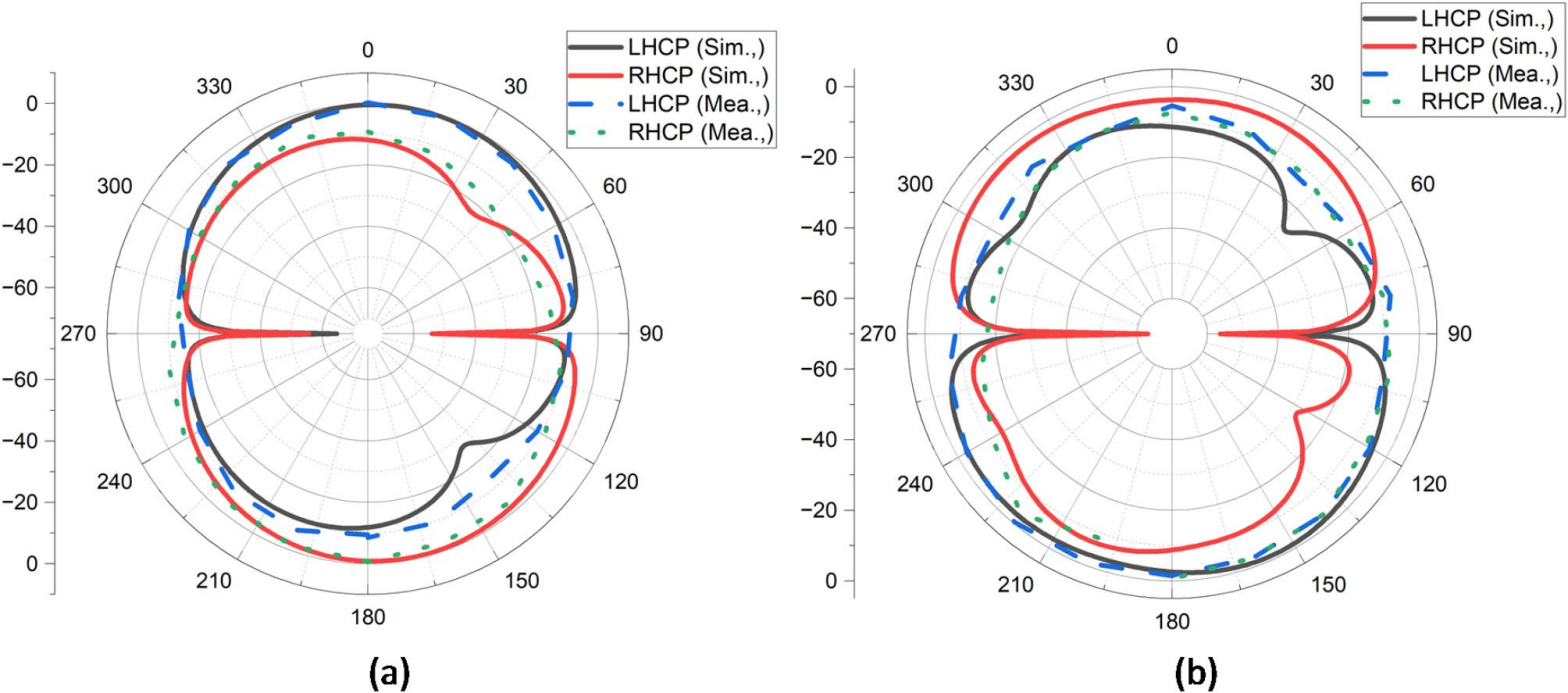
Far-field pattern of the proposed antenna (**a**) 2.5 GHz, (**b**) 6 GHz.

Figure 7 (a) and (b) show the far-field radiation patterns of the proposed antenna at 2.5 GHz and 6 GHz, highlighting both Left-Hand Circular Polarization (LHCP) and Right-Hand Circular Polarization (RHCP) for simulated and measured data. At 2.5 GHz, the radiation patterns for LHCP and RHCP are relatively symmetrical, with RHCP slightly more dominant. The measured results closely align with the simulations, though some discrepancies around 120° and 240° may be due to practical factors like manufacturing imperfections or environmental influences during measurement.

At 6 GHz, the RHCP pattern becomes more dominant, particularly around 90° and 270°, indicating that the antenna is more effective in producing right-hand circular polarization at this higher frequency. Overall, the antenna demonstrates consistent performance across the two frequencies, with stronger RHCP radiation at 6 GHz, validating its design for applications requiring robust circular polarization, particularly in higher frequency bands.

**Fig. 8:**
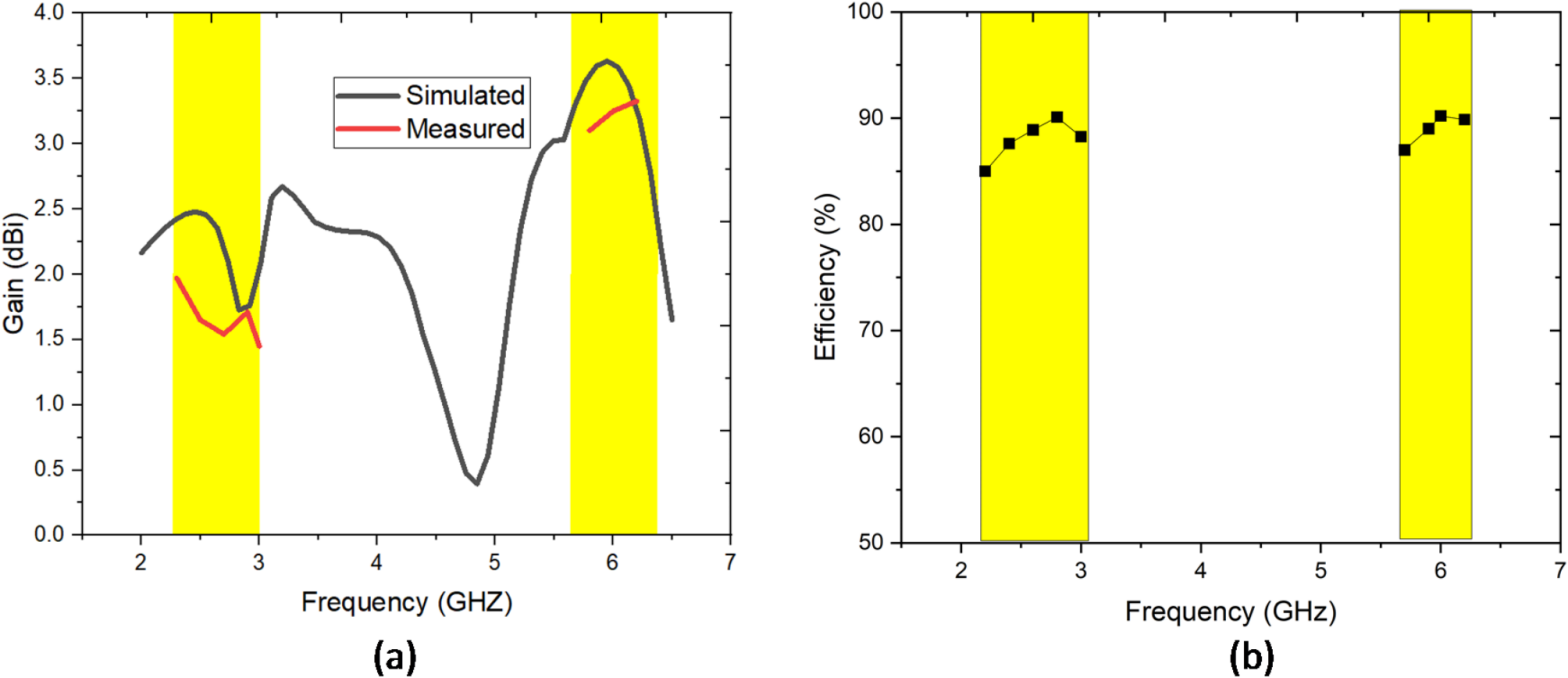
(**a**) Simulated and measured gain of the proposed antenna, (**b**) efficiency of the proposed antenna.

In the lower band (around 2 to 3 GHz), the simulated gain exhibits a peak near 2.5 dBi, while the measured gain is slightly lower, peaking around 1.8 dBi as shown in Fig. 8 (a). In the higher band (around 6 GHz), both simulated and measured gains align more closely, with the simulated gain reaching approximately 3.5 dBi and the measured gain slightly lower at 3.2 dBi. The lower-than-expected measured gain at lower frequencies may be attributed to practical factors such as fabrication tolerances, environmental conditions, or impedance mismatches. Figure 8 (b) illustrates the radiation efficiency of the proposed antenna across two operating frequency bands, specifically in the 2–3 GHz and 5.5–6.5 GHz ranges, highlighted by yellow regions. Within these bands, the efficiency remains consistently high, ranging between 85% and 90%, demonstrating the antenna’s effectiveness in converting input power into radiated energy with minimal losses. The efficiency peaks within the central regions of both frequency bands, indicating optimal performance at the intended operating frequencies. This stable and high efficiency across both bands is a crucial characteristic, showcasing the antenna’s suitability for dual-band applications while ensuring reliable and energy-efficient operation.

**Fig. 9:**
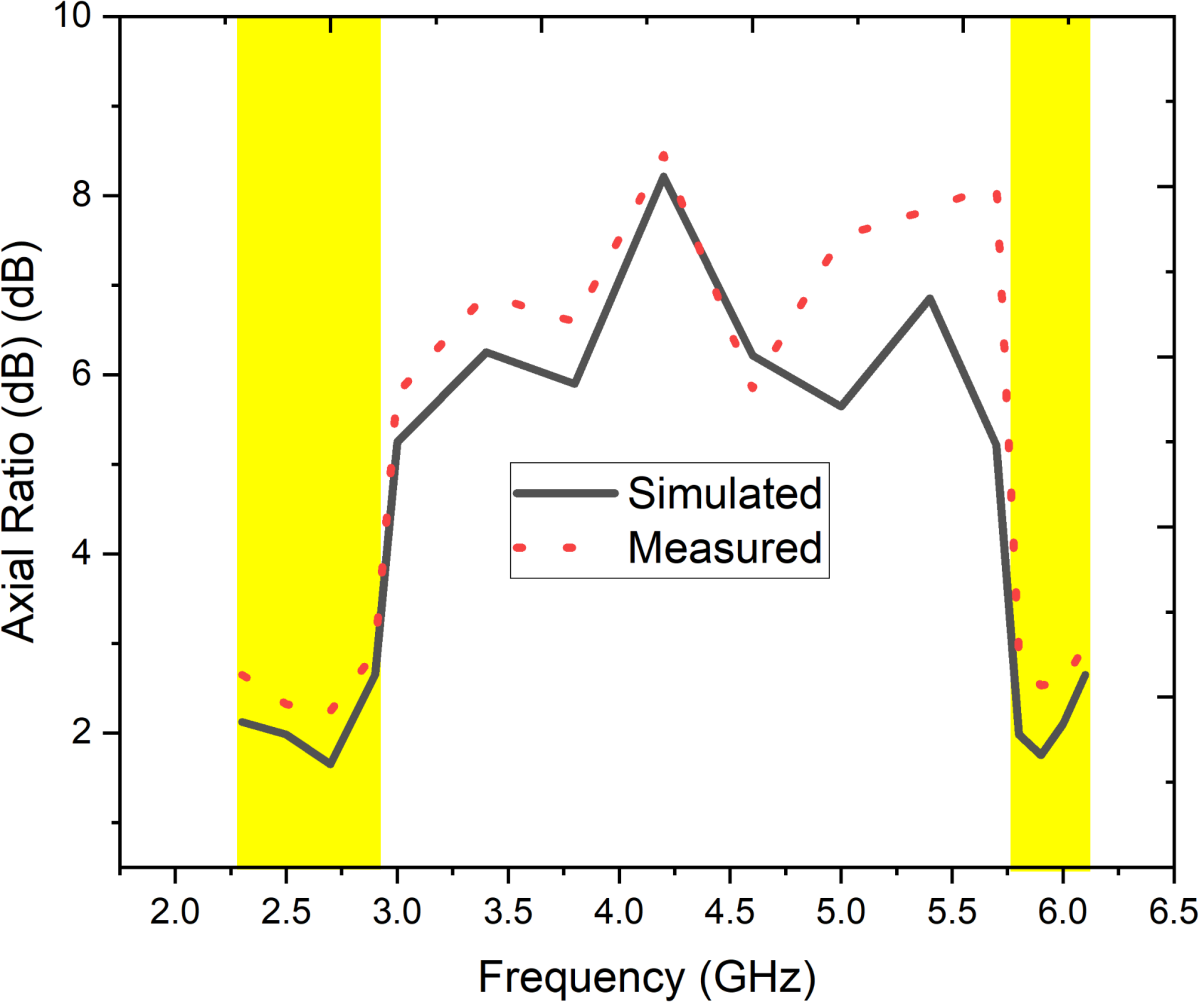
Simulated and measured Axial ratio of the proposed antenna.

The axial ratio (AR) plot presents the performance of the proposed antenna in terms of its ability to achieve circular polarization (CP) across two distinct frequency bands. Figure 9 compares the simulated (solid line) and measured (dashed red line) AR values. The simulated results indicate an AR minimum near 2.8 GHz and 5.8 GHz, with the AR dipping well below the 3 dB threshold, signifying excellent circular polarization performance at these frequencies. However, the measured data shows a slight deviation, particularly in the lower band (2.5–3.1 GHz), where the measured AR is higher than the simulated values, peaking around 2.6 GHz.

## Performance comparison

The proposed antenna outperforms existing designs in several key aspects as listed in Table 3. With a compact size of 0.26λ × 0.26λ, it is smaller than most designs in the literature, such as^10^ (0.45λ × 0.45λ) and^11^ (0.72λ × 0.72λ), while maintaining excellent performance. It supports dual-band operation at 2.5 GHz and 6 GHz, a feature not commonly addressed in many references. The proposed design also achieves a high efficiency of 85–90%, which is superior to designs like^11^ (57.5–60.25%) and comparable to high-efficiency antennas like^12^ (91–92%). Additionally, it offers a reasonable gain of 1.8 dBi and 3.25 dBi across its operating bands, with bandwidths of 23.08% and 6.78%, respectively, surpassing many other compact antennas. These attributes make the proposed design a highly efficient and compact solution for modern wireless applications.

**Table 3:** Performance comparison of the proposed antenna.

Ref.	Size of the antenna (λ^2^)	Ant. Type	Gain (dBi)	Cent. Freq. (GHz)	BW (%)	ARBW (%)	Efficiency (%)
^10^	0.45 × 0.45	Planar antenna	1.98/1.94	1.575/2.45	7.6/5.5	2.7	73.6/83
^11^	0.72 × 0.72	Monopole	6.6/7.2	3.5/5.8	11.7/9.1	2/8.2	57.5/60.25
^12^	0.4 × 0.35	Monopole	2.65/6.36	2.45/5.8	38.6/24.1	13.3/13.5	91/92
^19^	0.34 × 0.36	Cross-slot	3.4	1.565	34	20	Not given
^20^	0.83 × 1.67	Square patch	8.5	2.64	28	10.4	Not given
^21^	0.38 × 0.42	Monopole	2.7	2.3	4	4	Not given
^27^	0.4 × 0.4	Monopole	3	5.5	149	80.7	Not given
^30^	0.63 × 0.63	Circular patch	5.2	2.5	32.6	33	Not given
^31^	0.49 × 0.49	L-slot	2	1.66	30	32	90%
^35^	0.22 × 0.23	Square patch	3	2.4	8.3	1.35	Not given
Proposed work	0.5167 × 0.5167	Modified circular patch antenna	1.8 & 3.25	2.5 & 6	23.08 & 6.78	23.08 & 6.78	85–90%

## Potential application of the proposed antenna

**Fig. 10:**
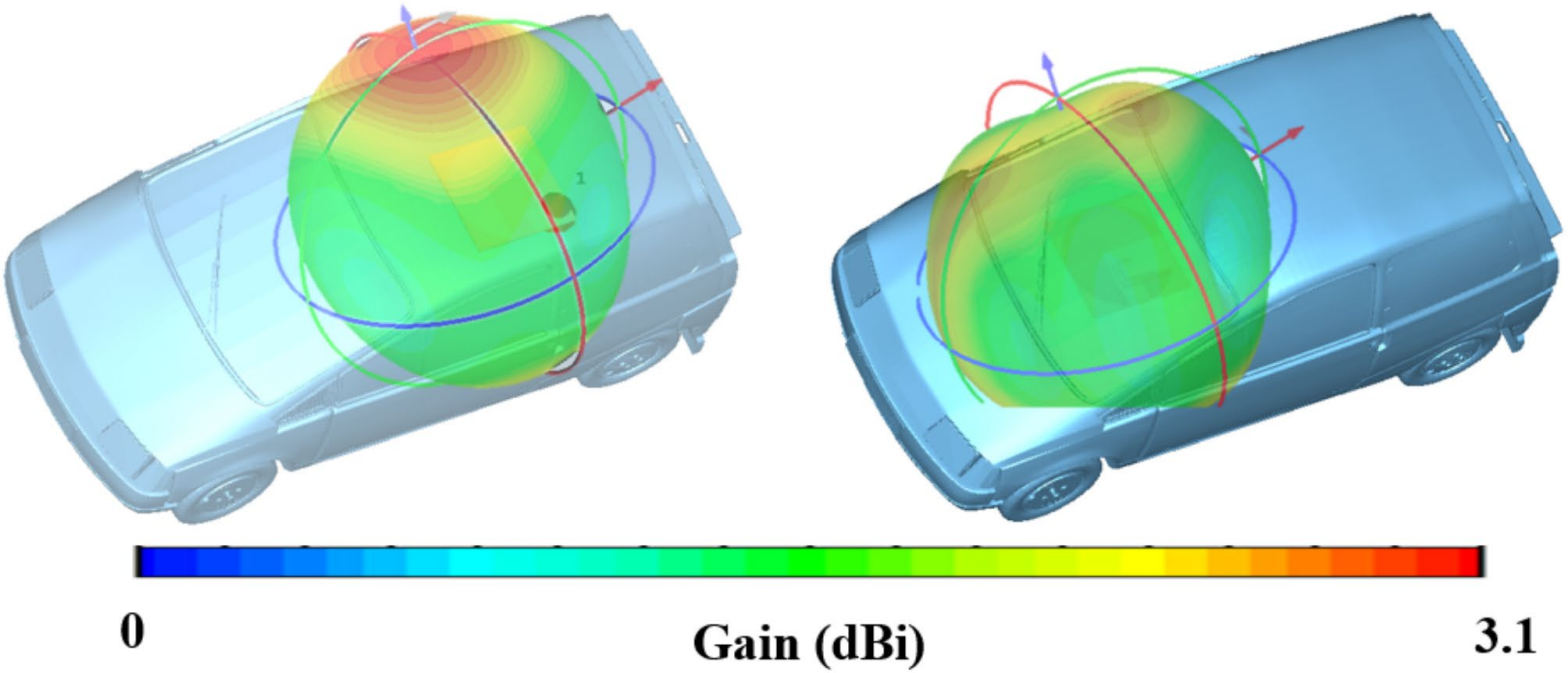
Placement analysis of the proposed antenna at 5.85 GHz.

Figure 10 illustrates the far-field radiation patterns of a proposed antenna operating at 5.85 GHz, analyzed in the context of vehicular placement. The two scenarios depicted involve positioning the antenna on the roof and near the front mirror of a vehicle to assess the impact of these placements on the antenna’s radiation performance. The 3D radiation patterns are overlaid on the vehicle model using CST Microwave Studio Suite 2021, providing a clear visual representation of the signal distribution around the car. The antenna exhibits omnidirectional radiation characteristics in both scenarios, with a near-spherical radiation pattern. This indicates that the antenna radiates energy uniformly in all directions, which is desirable for applications requiring consistent coverage around the vehicle. The comparison of the radiation patterns for the two placements reveals subtle differences in how the vehicle’s structure influences the antenna’s performance. The roof-mounted configuration seems to maintain a more symmetrical and broader radiation pattern, potentially offering better overall coverage. In contrast, the placement near the front mirror, while still providing substantial coverage, may experience slight distortions or reductions in gain due to the proximity to the car’s metallic structure, which could cause reflections or shadowing effects. The proposed antenna designs exhibit versatility and can be adapted for various advanced applications. For instance, they can be utilized in temperature and pressure sensing systems, drawing inspiration from Zhang et al.^44^, where slot antennas integrated with CSRR structures demonstrated excellent performance in high-temperature environments. Additionally, the antenna’s potential for target detection and positioning, as highlighted by Zhang et al.^45^, can be enhanced through integration with reconfigurable or holographic surfaces, enabling precise object localization for applications like surveillance and autonomous systems. Moreover, the designs align well with wearable technologies, as shown by Awan et al.^46^, where dual-band MIMO antennas with low mutual coupling supported efficient communication for 2.4/5.8 GHz wearable applications. These capabilities make the proposed antennas adaptable for modern, multifunctional use cases.

### Link budget analysis

The proposed antenna operates at dual frequencies of 2.5 GHz and 6 GHz, achieving a gain of 1.8 dBi and an effective isotropic radiated power (EIRP) of 13.985 dBm and 16.958 dBm, respectively, with a transmitted power of 10 dBm as listed in Table 4^49,50^. On the receiver side, the antenna gain is 5.98 dBi, and under standard ambient conditions (293 K), the noise power density is calculated as -198.974 dB/Hz, ensuring minimal noise interference. Supporting bit rates of 1, 10, 50, and 100 Mbps, the system maintains a bit error rate of 1 × 10⁻⁵ for reliable data communication. The ideal phase-shift keying (PSK) modulation requires an SNR of 9.56 dB, which is further enhanced by a coding gain of 4.8 dB, while a fixing deterioration of 2.125 dB accounts for practical implementation losses. These characteristics underscore the antenna’s suitability for robust and efficient wireless communication across multiple data rates.

**Table 4:** Various parameters considered for Link budget analysis for the proposed design.

Parameter	Value
Transmitter	
Operating frequency (GHz)	2.5/6
Antenna gain (dBi)	1.8 /2.5dBi
Transmitted power (dBm)	10
EIRP (dBm)	13.985/16.958
Receiver	
Receiver antenna gain (dBi)	5.98
Ambient temperature (K)	293
Boltzmann constant	1.38 × 10⁻²³
Noise power density (dB/Hz)	−198.974
Signal quality	
Bit rate (Mb/s)	1, 10, 50, 100
Bit error rate	1 × 10⁻⁵
Ideal PSK (dB)	9.56
Coding gain (dB)	4.8
Fixing deterioration (dB)	2.125

**Fig. 11:**
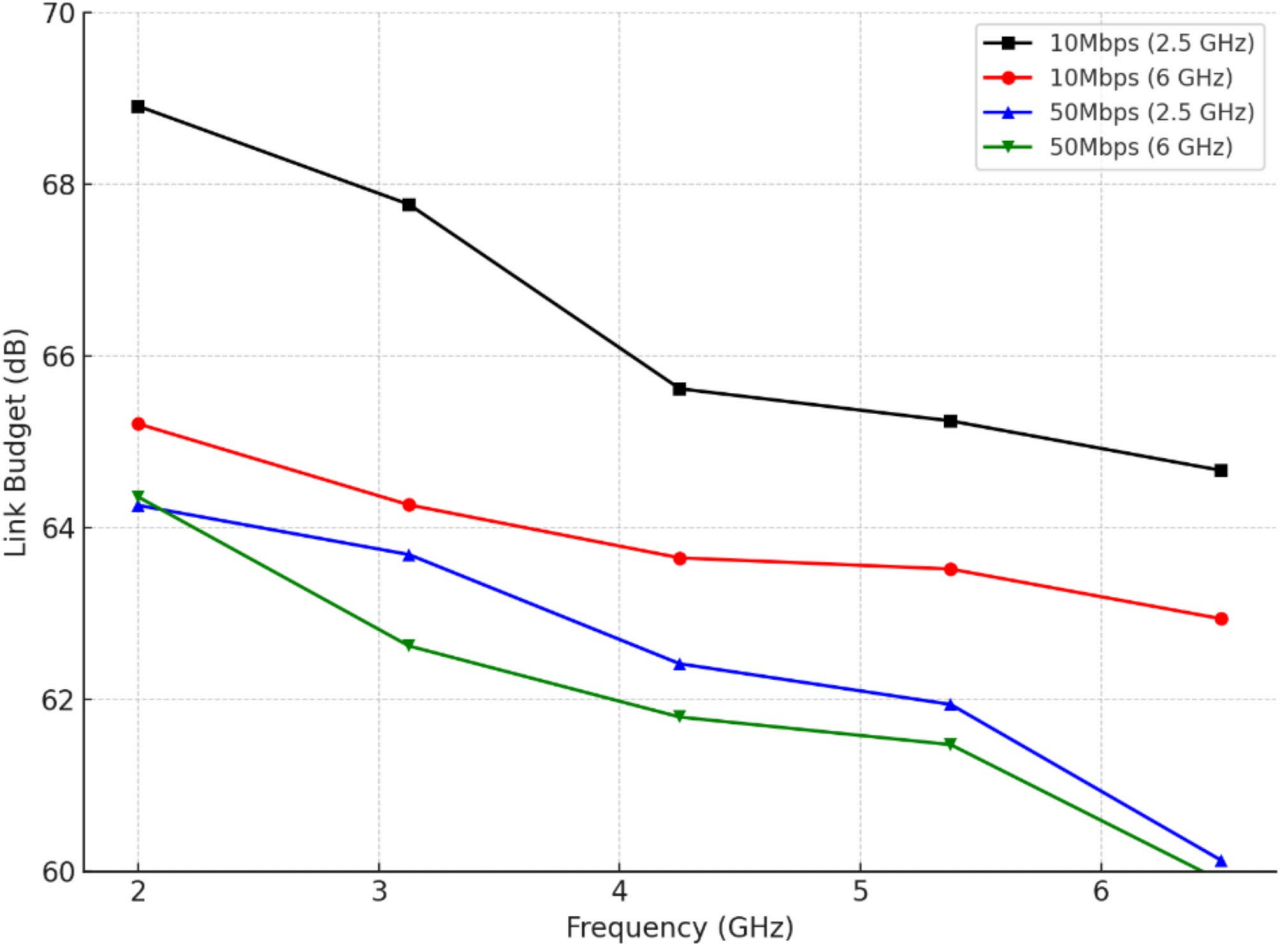
Frequency vs. link budget of the proposed antenna.

Figure 11 shows the variation in link budget as a function of frequency for different data rates and operating frequencies. It includes four curves: two corresponding to a data rate of 10 Mbps at 2.5 GHz and 6 GHz, and two for a data rate of 50 Mbps at the same frequencies. The link budget, measured in decibels (dB), is plotted across a frequency range from 2 GHz to 6 GHz. For the 10 Mbps case, the link budget at 2.5 GHz is consistently higher than at 6 GHz, indicating better performance at lower frequencies. Similarly, for the 50 Mbps case, the link budget at 2.5 GHz exceeds that at 6 GHz. In all scenarios, the link budget decreases as frequency increases, reflecting higher path loss and signal attenuation at higher frequencies. Additionally, higher data rates (50 Mbps) exhibit lower link budgets compared to lower data rates (10 Mbps) at the same frequency, highlighting the trade-off between data rate and communication performance.

## Conclusion

The proposed antenna, designed for dual-band operation at 2.5 GHz and 6 GHz, has demonstrated its effectiveness through both simulation and measurement. With compact dimensions of 62 mm × 62 mm × 1.6 mm, corresponding to 0.5167λ × 0.5167λ × 0.0133λ.at 2.5 GHz, the antenna offers a low-profile solution, making it ideal for space-constrained applications. The measured results align well with the simulated data, confirming axial ratios below 3 dB for both operating bands, which ensures stable circular polarization. The antenna also exhibits peak gains of 1.8 dBi at the lower band and 3.8 dBi at the higher band, further validating its performance. The antenna, which employs a modified circular patch and a novel feeding element on a 1.6 mm thick FR4 substrate, meets the stringent demands of modern wireless communication systems, particularly in Industrial, Scientific, and Medical (ISM) and Vehicle-to-Everything (V2X) applications. Its ability to provide efficient radiation and circular polarization across two frequency bands makes it a versatile solution for dual-band communication systems. Overall, the compact design and reliable performance make this antenna a strong candidate for deployment in next-generation communication technologies.

## Data Availability

All data generated or analysed during this study are included in this published article. Data will be made available on request from D. Rajesh Kumar.
